# SspABCD-SspFGH Constitutes a New Type of DNA Phosphorothioate-Based Bacterial Defense System

**DOI:** 10.1128/mBio.00613-21

**Published:** 2021-04-27

**Authors:** Shiwei Wang, Mengping Wan, Ruolin Huang, Yujing Zhang, Yuqing Xie, Yue Wei, Mustafa Ahmad, Dan Wu, Yue Hong, Zixin Deng, Shi Chen, Zhiqiang Li, Lianrong Wang

**Affiliations:** a Key Laboratory of Combinatorial Biosynthesis and Drug Discovery, Ministry of Education, School of Pharmaceutical Sciences, Wuhan University, Wuhan, Hubei, China; b Taihe Hospital, Hubei University of Medicine, Shiyan, Hubei, China; c Department of Burn and Plastic Surgery, Division of Wound Repair, Shenzhen Institute of Translational Medicine, The First Affiliated Hospital of Shenzhen University, Shenzhen Second People’s Hospital, Shenzhen, China; d Department of Neurosurgery, Zhongnan Hospital, Wuhan University, Wuhan, Hubei, China; Northern Arizona University

**Keywords:** DNA phosphorothioate modification, SspFGH defense, phage resistance, additive defense

## Abstract

Unlike nucleobase modifications in canonical restriction-modification systems, DNA phosphorothioate (PT) epigenetic modification occurs in the DNA sugar-phosphate backbone when the nonbridging oxygen is replaced by sulfur in a double-stranded (ds) or single-stranded (ss) manner governed by DndABCDE or SspABCD, respectively. SspABCD coupled with SspE constitutes a defense barrier in which SspE depends on sequence-specific PT modifications to exert its antiphage activity. Here, we identified a new type of ssDNA PT-based SspABCD-SspFGH defense system capable of providing protection against phages through a mode of action different from that of SspABCD-SspE. We provide further evidence that SspFGH damages non-PT-modified DNA and exerts antiphage activity by suppressing phage DNA replication. Despite their different defense mechanisms, SspFGH and SspE are compatible and pair simultaneously with one SspABCD module, greatly enhancing the protection against phages. Together with the observation that the *sspBCD-sspFGH* cassette is widely distributed in bacterial genomes, this study highlights the diversity of PT-based defense barriers and expands our knowledge of the arsenal of phage defense mechanisms.

## Abstract (precis)

We recently found that SspABCD, catalyzing single-stranded (ss) DNA phosphorothioate (PT) modification, coupled with SspE provides protection against phage infection. SspE performs both PT-simulated NTPase and DNA-nicking nuclease activities to damage phage DNA, rendering SspA-E a PT-sensing defense system.

## INTRODUCTION

To proliferate in phage-rich environments, prokaryotes have developed a diverse array of defense machinery and tactics that target every stage of phage infection. Among these are well-known phage adsorption-blocking mechanisms (1), restriction-modification (R-M) systems (2), abortive infection systems (3), toxin-antitoxin mechanisms, and clustered regularly interspaced short palindromic repeat (CRISPR)-CRISPR-associated (Cas) systems (4–6). Considering the diversity of phages and the recent discovery of new defense systems such as BREX (7, 8), DISARM (9), Zorya, and Wadjet (10), there is no doubt that our understanding of the arsenal of defense measures is insufficient, and many phage-resistance systems residing in prokaryotic genomes remain to be explored. Indeed, this view is reinforced by our discovery of DNA phosphorothioate (PT) modification-based defense barriers, which provide protection against genetic parasites by virtue of the self/nonself discrimination principle, somewhat resembling methylation-based R-M systems (11–13). However, unlike DNA nucleobase modifications in R-M systems, e.g., *N*6-methyl-adenine, *N*4-methyl-cytosine, *C*5-methyl-cytosine, and 7-deazaguanine derivatives, DNA PT modification occurs in the DNA sugar-phosphate backbone with the nonbridging oxygen replaced by sulfur (2, 14, 15).

To date, DNA PT modification has been found to occur in a sequence-selective and *R*_P_ configuration-specific manner governed by DndABCDE or SspABCD in a wide range of bacterial and archaeal strains (12, 14). Typically, *sspBCD* and *dndBCDE* are present in the form of a three- and four-gene operon, respectively. However, the functions of both SspA and DndA as cysteine desulfurases can be performed instead by an IscS ortholog, which is consistent with the distribution of *sspA* and *dndA* either adjacent to *sspBCD* and *dndBCDE*, respectively, or elsewhere in some prokaryotic genomes (12, 16, 17). DndABCDE-mediated DNA PT modifications, typically double stranded (ds), occur in 4-bp consensus motifs, e.g., 5′-G_PS_AAC-3′/5′-G_PS_TTC-3′ in Salmonella enterica serovar Cerro 87 and Escherichia coli B7A, 5′-G_PS_GCC-3′/5′-G_PS_GCC-3′ in Pseudomonas fluorescens pf0-1, and 5′-G_PS_ATC-3′/5′-G_PS_ATC-3′ in Hahella chejuensis KCTC 2396 (PS stands for phosphate-sulfur linkage) (18–20). In contrast, SspABCD catalyzes single-stranded (ss) DNA PT modification at 5′-C_PS_CA-3′; no PT modification is found in 5′-TGG-3′ on the complementary strand (12, 18). Notably, the functional similarities between DndA and SspA as cysteine desulfurases and between DndC and SspD as ATP pyrophosphatases lead to the prediction that the DndABCDE and SspABCD machineries share the initial sulfur mobilization pathway but follow divergent DNA target selection steps (12). This divergence is considered to occur due to SspB, a DNA-nicking nuclease whose nicking activity has been demonstrated to be essential to ssDNA PT formation but has not yet been found for DndABCDE proteins (12).

In parallel with PTs with different characteristics, Dnd and Ssp systems provide protection against foreign DNA by different mechanisms. In the Dnd system, DndABCDE constitutes a defense barrier with the 3-gene product DndFGH, in which DndFGH employs sequence-specific PT modification as a recognition tag to distinguish and destroy invasive non-PT-modified DNA (13). Upon the loss of PTs, host genomes undergo self-restriction by unrestrained DndFGH, which triggers the cellular SOS response (21, 22). This is in sharp contrast to the PT-sensing defense mechanism of the Ssp system, in which the restriction component SspE relies on SspABCD-mediated ssDNA PT modification to exert its antiphage activity (12). This is attributed to the activation of the GTPase activity of SspE, which is essential to its defensive function, by PT modification at 5-C_PS_CA-3′ in the host DNA (12). Notably, both types of DNA PT modifications exhibit unusual modification features: (i) only 10% to 15% of the genome-wide 5′-CCA-3′ motifs in FF75 and 5′-GAAC-3′/5′-GTTC-3′ in B7A and Cerro 87 are detected as PT protected, and (ii) PT profiles are heterologous in a population of DNA molecules even in the presence of the active restriction counterpart DndFGH or SspE (18, 23, 24). These features distinguish PT-based defense barriers from classical methylation-based R-M systems and suggest unusual target selection for both the PT modification and restriction components.

Here, we report that the PT-modifying genes *sspBCD* and *iscS* in Vibrio anguillarum strain FF-93 can provide protection against a range of phages in combination with *sspFGH*, a conserved three-gene module sharing no sequence homology with SspE or DndFGH. SspFGH employs ssDNA PT modification as a recognition tag to introduce damage to non-PT-modified phage genomes and consequently impair phage DNA replication. Unrestrained SspFGH exhibits toxicity to PT-lacking cells, suggesting a different defense mechanism from that of SspE. However, SspFGH and SspE are compatible in a single *sspBCD*-containing cell and confer an additive level of phage resistance. Considering the wide distribution of PT-based SspFGH defense modules in bacteria, our study highlights the diversity of the gene contents and modes of action of PT-based defense systems and expands our understanding of the arsenal of bacterial defensive measures.

## RESULTS

### A new type of PT-based SspFGH antiphage barrier.

**FIG 1:**
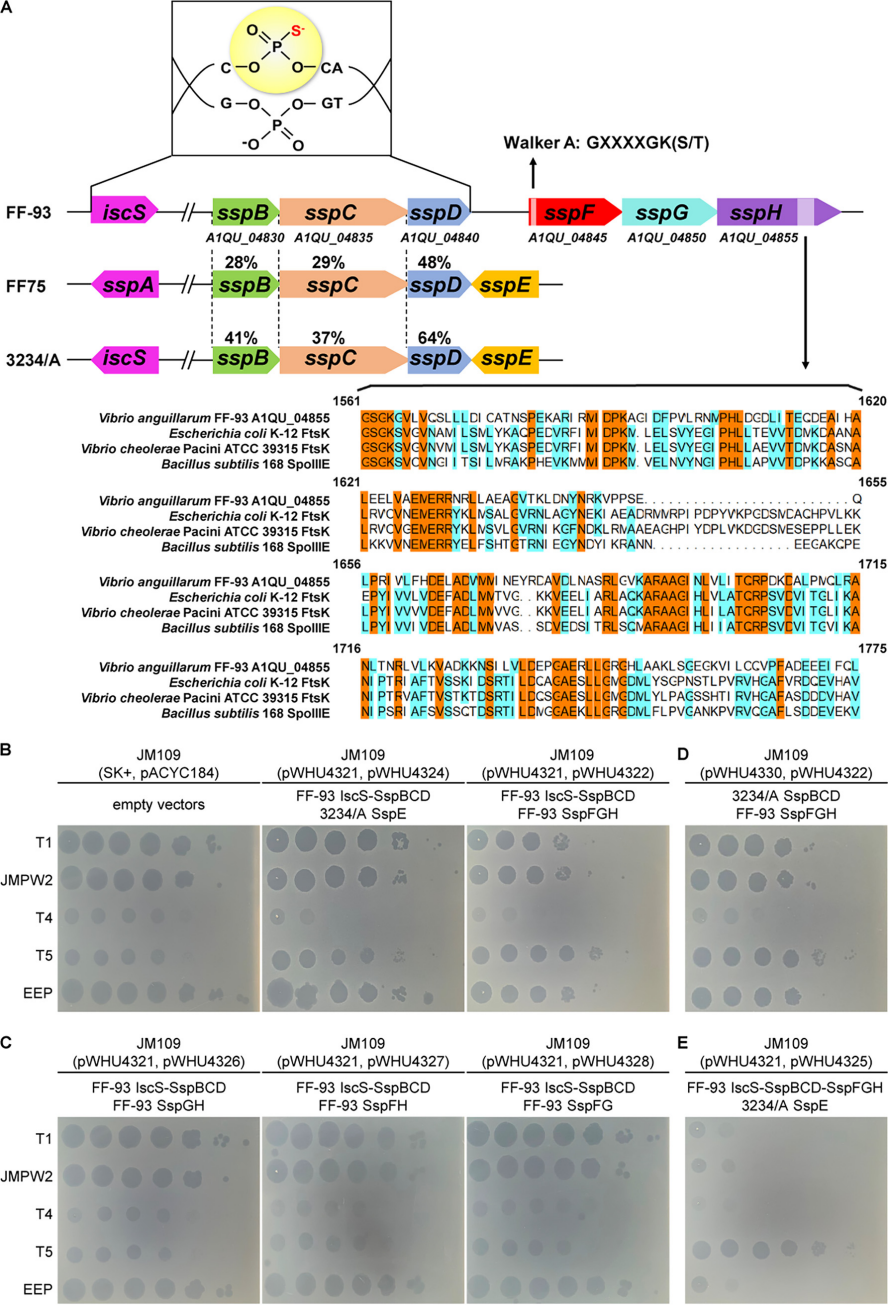
Genetic organization of genes involved in the phosphorothioate-based SspFGH defense system. (A) Comparison of PT-based SspFGH and SspE modules in V. anguillarum FF-93, *V. cyclitrophicus* FF75, and E. coli 3234/A. PT modification at the 5′-C_PS_CA-3′ motif conferred by IscS and SspBCD in FF-93 is schematically shown. The numbers between dotted lines are the percentages of amino acid sequence identity between the corresponding Ssp proteins. The C-terminal region of SspH was aligned with FstK from Escherichia coli K-12 (UniProtKB identifier [ID] P46889) and Vibrio cholerae Pacini ATCC 39315 (UniProtKB ID Q84I33) and SpoIIIE from Bacillus subtilis 168 (UniProtKB ID P21458). Identical amino acid residues in all four sequences are shaded in yellow, and similar amino acids are in cyan. (B to D) Two microliters of 10-fold serial dilutions of phage indicated on the left were spotted onto a lawn of E. coli JM109 possessing empty vectors or different combinations of *ssp* genes as indicated.

SspB, SspC, and SspD in Vibrio anguillarum FF-93 (GenBank accession number [no.] AJYT00000000.2) share 28% to 64% amino acid sequence identity with those in Vibrio cyclitrophicus FF75 and Escherichia coli 3234/A (Fig. 1A). When coupled with SspE from 3234/A, *sspBCD* and *iscS* in the pBluescript II SK(+)-derived plasmid pWHU4321 conferred resistance to a group of phages (T1, JMPW2, T4, and EEP) on the heterologous E. coli host JM109, although the resistance amounted to only 1 to 2 orders of magnitude (Fig. 1B and Table 1). Together with the detection of PT-linked d(C_PS_C) dinucleotides by liquid chromatography-coupled tandem quadrupole mass spectrometry (LC-MS/MS) in JM109(pWHU4321) (see Fig. S1 in the supplemental material), the results demonstrated that IscS and SspBCD in FF-93 also confer DNA PT modification at 5′-C_PS_CA-3′ consensus sequences capable of stimulating the antiphage defense of SspE from 3234/A. Although no trace of SspE was detected in the FF-93 genome, a three-gene cassette consisting of *A1QU_04845*, *A1QU_04850*, and *A1QU_04855* was found adjacent to the *sspBCD* cluster (Fig. 1A). The protein-coding regions of these genes showed no significant sequence homology to annotated genes except for an ATP-binding Walker A motif (GXXXXGK[S/T], where X is any residue) in A1QU_04845 and some similarities of the C-terminal end of A1QU_04855 to the sequences of reported FtsK/SpoIIIE family proteins (Fig. 1A).

**TABLE 1:** Effects of PT-based SspFGH system on phage infectivity

Phage	Efficiency of plaquing for E. coli JM109 harboring:			
	pWHU4321, pWHU4324 (FF-93 IscS-SspBCD 3234/A SspE)	pWHU4321, pWHU4322 (FF-93 IscS-SspBCD, FF-93 SspFGH)	pWHU4330, pWHU4322 (3234/A SspBCD FF-93 SspFGH)	pWHU4321, pWHU4325 (FF-93 IscS-SspBCD-SspFGH 3234/A SspE)
T1	(2.0 ± 0.0) × 10^−1^	(2.8 ± 0.1) × 10^−2^	(3.3 ± 0.2) × 10^−2^	(8.6 ± 0.4) × 10^−5^
JMPW2	(1.7 ± 0.1) × 10^−1^	(2.2 ± 0.1) × 10^−2^	(2.4 ± 0.0) × 10^−2^	(5.3 ± 0.2) × 10^−4^
T4	(1.9 ± 0.1) × 10^−2^	(2.4 ± 0.2) × 10^−3^	(2.9 ± 0.0) × 10^−3^	(2.5 ± 0.2) × 10^−4^
T5	(1.1 ± 0.1) × 10^0^	(1.1 ± 0.1) × 10^0^	(1.1 ± 0.1) × 10^0^	(1.0 ± 0.1) × 10^0^
EEP	(1.0 ± 0.2) × 10^−1^	(8.3 ± 0.2) × 10^−2^	(6.2 ± 0.1) × 10^−2^	(4.6 ± 0.5) × 10^−4^

10.1128/mBio.00613-21.1 FIG S1 LC-MS/MS detection of PT-modified d(C_PS_C) dinucleotides. The heterologous expression of *iscS* and *sspBCD* from FF-93 on pWHU4321 resulted in PT-modified d(C_PS_C) in E. coli JM109. No d(C_PS_C) was detected in E. coli JM109 lacking *ssp* genes. The fragmentation pattern of a d(C_PS_C) standard is shown in the structural inset. Download FIG S1, TIF file, 1.8 MB. Copyright © 2021 Wang et al. 2021 Wang et al. https://creativecommons.org/licenses/by/4.0/ This content is distributed under the terms of the Creative Commons Attribution 4.0 International license.

Given that *A1QU_04845*, *A1QU_04850*, and *A1QU_04855* were cotranscribed as a single operon and their positions relative to the *sspBCD* operon (see Fig. S2 in the supplemental material), we speculated that this three-gene cluster constitutes a possible new type of PT-based phage defense system. To test this hypothesis, we cloned the *A1QU_04845*-*04850-04855* locus into pACYC184, and the resultant plasmid pWHU4322 was transformed into IscS-SspBCD-expressing JM109(pWHU4321). When challenged with phages, including T1, JMPW2, T4, T5, and EEP, JM109(pWHU4321, pWHU4322) cells exhibited resistance to all phages tested (except phage T5), demonstrating the involvement of *A1QU_04845* to *A1QU_04855* in defensive function (Fig. 1B). Moreover, deletion of *A1QU_04845*, *A1QU_04850*, or *A1QU_04855* abolished the protection against phage infection, suggesting the essential role of each of the three genes, which are here denoted *sspF*, *sspG*, and *sspH*, respectively (Fig. 1A and C). To quantify the protection, we measured the phage efficiency of plaquing (EOP) on *iscS-sspBCD-SspFGH*-expressing JM109(pWHU4321, pWHU4322) versus that of *ssp*-lacking control cells. SspFGH provided moderate levels of protection against phages, with 3 orders of magnitude of protection against phage T4 and 2 orders of magnitude against phages T1, JMPW2, and EEP (Table 1). Collectively, these results suggested that SspFGH coupled with IscS-SspBCD behaves as a new type of ssDNA PT modification-based defense barrier to fend off phage invasion.

10.1128/mBio.00613-21.2 FIG S2 RT-PCR analysis of the cotranscription of *sspFGH* in V. anguillarum FF-93. Primers are schematically displayed above or below the *ssp* genes. PCR products were generated using reverse-transcribed cDNA (lanes 2, 5, and 8), nontranscribed mRNA (lanes 3, 6, and 9) and genomic DNA (gDNA) (lanes 4, 7, and 10) of FF-93 as the templates. Primer A (5′-GAATTTCGCCTTGGGAACCGACG-3′) and primer B (5′-GTCGGGCGAATGAATCAAAACG-3′) were used as the primer pair to produce 700-bp PCR fragments, primer C (5′-CTCCCTCTGGTGATCCGGATAC-3′) and primer D (5′-GCACGGCACGTATACCTTCCGG-3′) were used to generate 692-bp fragments, and primers A and D were used to yield 2,136-bp products. Lane 1, DNA marker. Download FIG S2, TIF file, 2.7 MB. Copyright © 2021 Wang et al. 2021 Wang et al. https://creativecommons.org/licenses/by/4.0/ This content is distributed under the terms of the Creative Commons Attribution 4.0 International license.

### SspFGH disturbs phage DNA replication.

**FIG 2:**
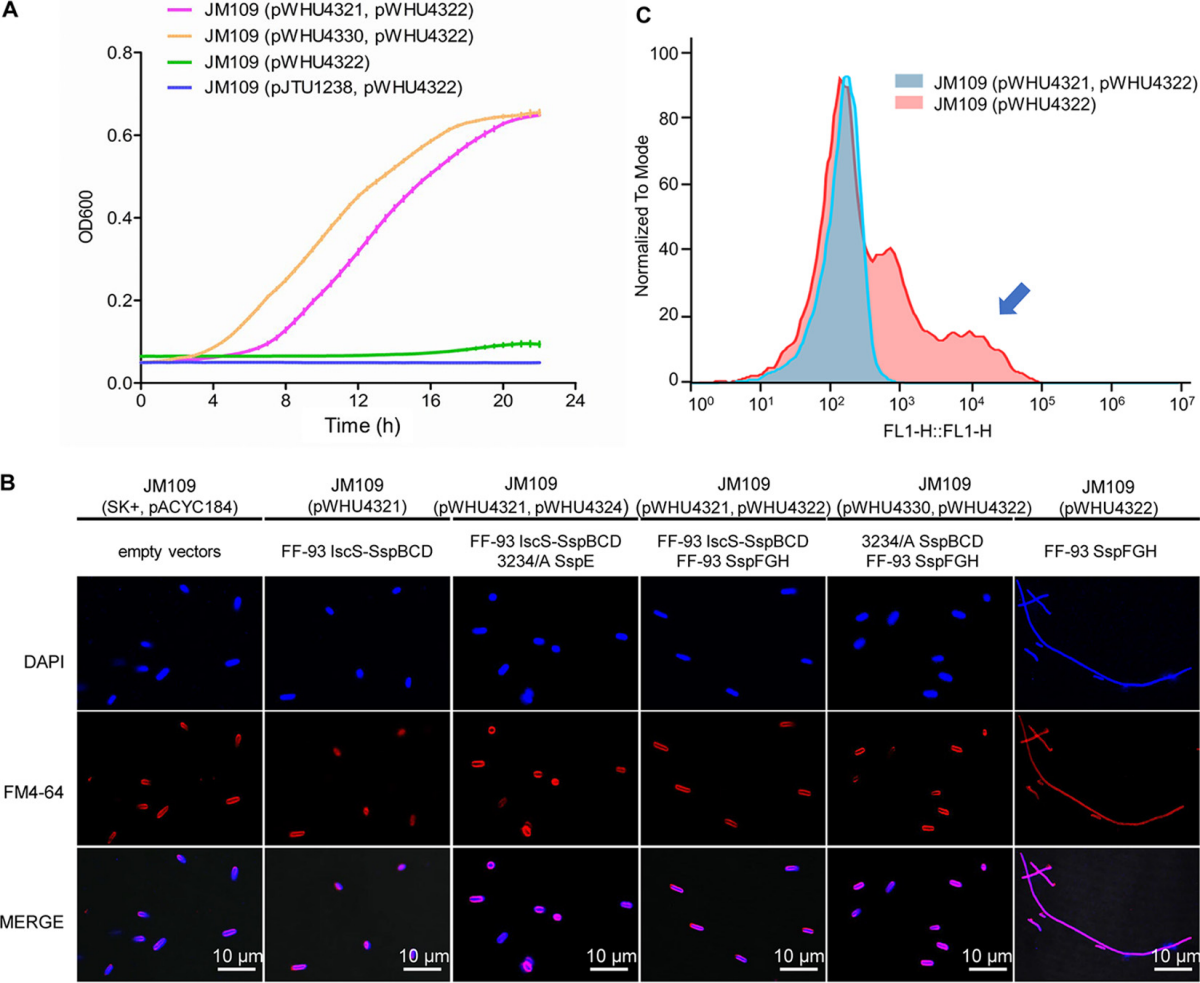
Effects of SspFGH and its derivatives on cell growth and morphology. (A) Growth impairment caused by SspFGH of FF-93 was resolved by complementation of the JM109(pWHU4322) strain with plasmid pWHU4321 or pWHU4330 expressing *iscS-sspBCD* from FF-93 or *sspBCD* from E. coli 3234/A, respectively, rather than pJTU1238 expressing the *dndBCDE* dsDNA PT modification gene cluster from S. enterica serovar Cerro 87. (B) Cell morphology of JM109 cells expressing different sets of *ssp* genes. Cell elongation was observed in SspFGH-expressing PT-lacking JM109(pWHU4322). (C) TUNEL assay analysis revealed that TUNEL-positive JM109(pWHU4322) cells resulted from SspFGH-induced DNA damage.

**FIG 3:**
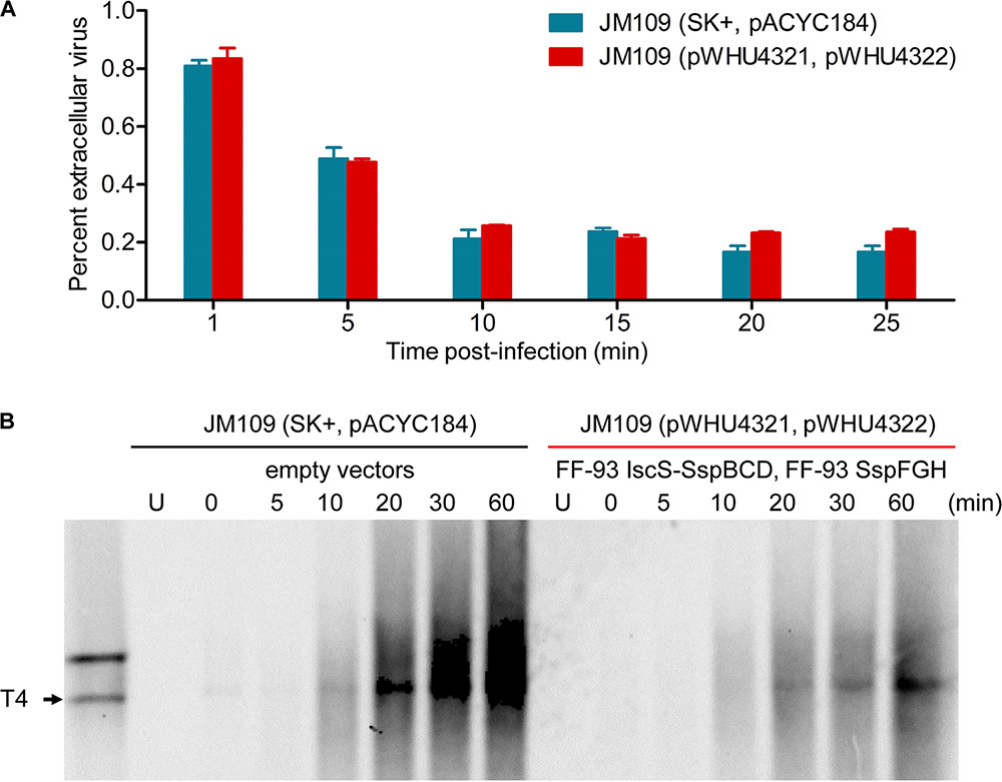
Adsorption and replication of phage T4 in JM109 cells. (A) Adsorption assay measured the percentage of extracellular free phage T4 remaining over a 25-min period. PT-based SspFGH defense in JM109(pWHU4321, pWHU4322) does not interfere with phage adsorption. (B) Southern blot analysis of the phage T4 genome in infected JM109 cells containing PT-based SspFGH or empty vectors over a 60-min period. The probe was designed to match region 148,913 to 149,433 in the T4 genome. Each lane contained 2 μg of total intracellular DNA digested with PacI or 1.5 μg of undigested total DNA. U, uninfected.

To understand the mechanism of action of SspFGH in phage defense, we first undertook studies to evaluate the effects of its expression in JM109 cells. The immediate observation was that SspFGH alone was detrimental to PT-lacking JM109(pWHU4322) cells, causing apparent growth impairment and a cell filamentation phenotype, which was in sharp contrast to the PT-dependent defense of SspE (Fig. 2A and B). However, this result was reminiscent of the DNA damage-induced SOS response arising from unrestrained DndFGH in PT-deficient S. enterica mutants (21, 22), inspiring us to assess the DNA integrity in SspFGH-expressing PT-lacking JM109(pWHU4322) cells by the terminal deoxyribonucleotide transferase (TdT)-mediated dUTP nick end labeling (TUNEL) assay. In this approach, the 3′-OHs of DNA damages are enzymatically labeled with fluorescein isothiocyanate (FITC)-labeled dUTP, resulting in increased cellular fluorescence values measurable by flow cytometry. Figure 2C shows that in the absence of the PT-modifying component *iscS-sspBCD*, the expression of SspFGH in JM109(pWHU4322) cells resulted in a TUNEL-positive cell population. In combination with the observation that SspFGH did not block phage adsorption (Fig. 3A), these results showed that the PT-based SspFGH barrier exerts antiphage activity by introducing damage to non-PT-modified phage genomes.

To verify this prediction, we monitored the replication of phage T4 DNA within infected cells during a phage infection time course. At successive time points following infection, total DNA, including chromosomal DNA and intracellular phage DNA, was isolated from T4-infected JM109 cells, digested with PacI, and subjected to gel electrophoresis and then to Southern hybridization experiments as previously described (12). In sharp contrast to the extensive replication of T4 DNA in *ssp*-lacking JM109 cells, its DNA replication was markedly suppressed in the presence of IscS-SspBCD-SspFGH (Fig. 3B). This result verified that SspFGH exerts a defensive function by inhibiting phage DNA replication. Surprisingly, no apparent DNA degradation debris was detected in Southern hybridization, a phenomenon seemingly inconsistent with the observed DNA damage arising from SspFGH in the PT-lacking JM109(pWHU4322) cells by the TUNEL assay (Fig. 2C). One plausible explanation is that in parallel with molecule-to-molecule PT heterogeneity, SspFGH does not consistently attack invasive phage DNA molecules at a given site in a population of infected cells, leading to highly diversified phage DNA damage patterns and the absence of specific DNA breakage products in Southern blotting.

### SspFGH and SspE are compatible, and their combination confers additive protection against phages.

Given the distinct mechanism of action of SspFGH and SspE, we asked whether PTs in the SspABCD-SspE system can protect DNA from the restriction of SspFGH. To answer this question, we cotransformed JM109 cells with pWHU4330 and pWHU4322, expressing the hybrid PT-modifying component SspBCD from the *sspB-E* module of E. coli 3234/A and SspFGH from FF-93, respectively. Interestingly, PT-modified DNA from the SspBCD-SspE system was also recognized as self-DNA by SspFGH based on two observations: (i) the growth impairment caused by unrestrained SspFGH in JM109(pWHU4322) was resolved by complementation with pWHU4330, and (ii) JM109(pWHU4330, pWHU4322) exhibited resistance to phages (Fig. 1D and 2). In contrast, the toxicity of SspFGH was not counteracted by double-stranded 5′-G_PS_AAC-3′/5′-G_PS_TTC-3′ PT modification mediated by DndBCDE from S. enterica serovar Cerro 87 on pJTU1238, suggesting that the sequence-specific DNA PT modification is critical for the self/nonself discrimination of SspFGH (Fig. 2A). Given that DNA PT modification is heterologous in a population of cells, we speculate that in addition to PT in the consensus sequences, more information is considered to enable SspFGH to distinguish self-DNA from non-PT-modified foreign DNA, e.g., the density of PTs along DNA molecules (24).

Considering that both SspFGH and SspE can pair with 5′-C_PS_CA-3′ PT modification to exert antiphage functions, we next set out to examine their compatibility and the effectiveness of their defenses when they are combined in a single strain. When coexisting in a single IscS-SspBCD-expressing JM109(pWHU4321, pWHU4325) strain, SspFGH from FF-93 and SspE from 3234/A were compatible and provided a greatly enhanced level of protection of up to 5 orders of magnitude against phage T1 (Fig. 1E and Table 1). Compared to the protection by SspFGH (EOP of 10^−2^ to 10^−3^) or SspE (EOP of 10^−1^ to 10^−2^) alone, the data indicated an additive effect of the two defense mechanisms to increase the overall phage resistance of a bacterial host (Table 1).

### SspBCD-SspFGH module is widespread in bacteria.

**FIG 4:**
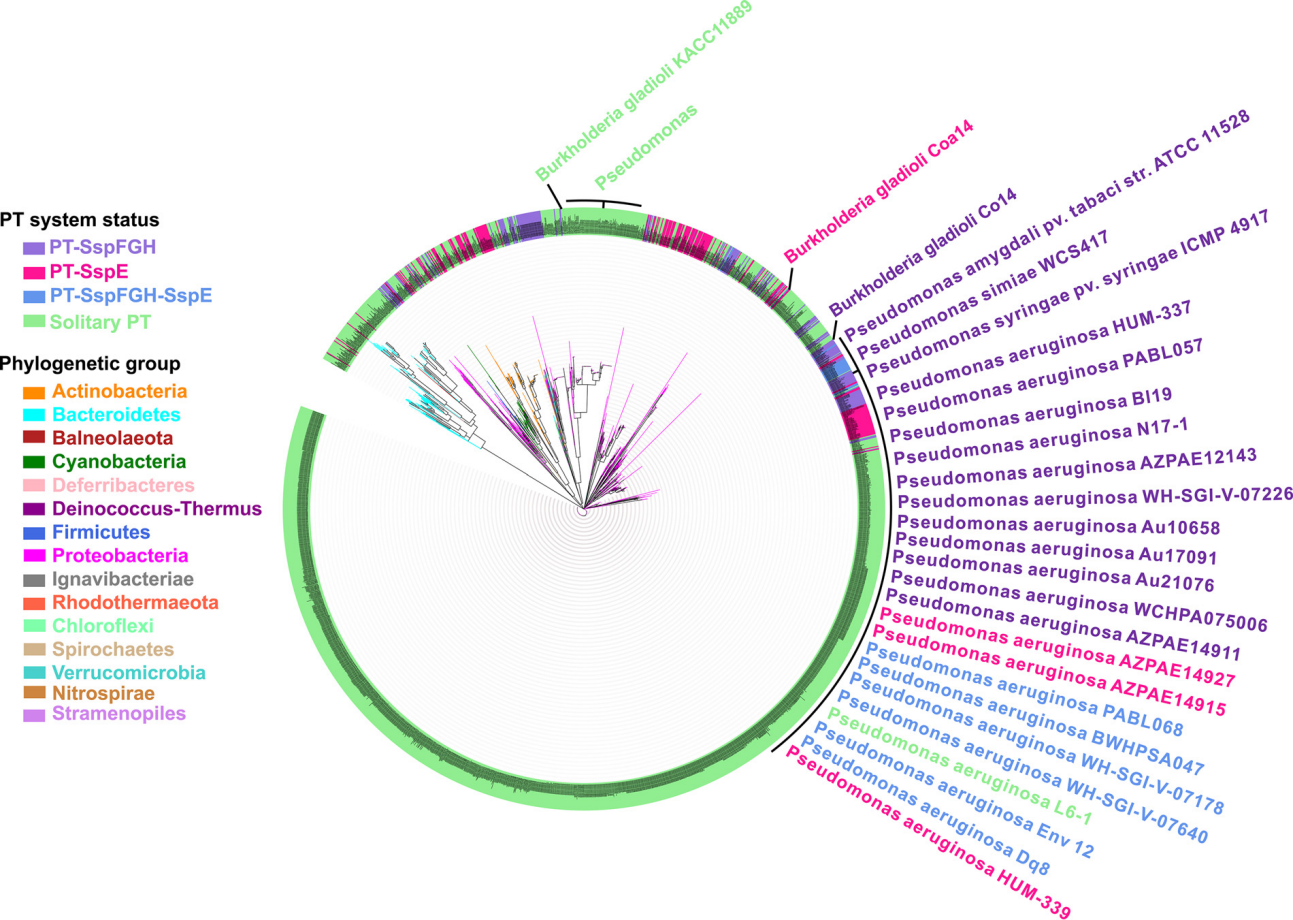
Phylogenetic distribution of SspBCD homologs. The phylogenetic tree of SspBCD in 2,678 strains was built using MEGA X 11.0 with different genera colored accordingly. The strains shaded purple, pink, blue, and green indicate that SspBCD is accompanied by SspFGH, SspE, both, or none, respectively. For clarity, strain names are also provided in Table S2 in the supplemental material.

After establishing the antiphage activity of SspFGH, we set out to investigate the phylogenetic distribution of SspFGH-mediated defense barriers in bacterial genomes. Using the well-characterized SspBCD amino acid sequences of FF75 as queries to perform a BLAST search against the NCBI databases (including the nonredundant nucleotide collection, reference sequence genome database, genomic survey sequences, high-throughput genomic sequences, and NCBI Genome) as previously described (12, 20), we found a total of 2,678 occurrences of *sspBCD* in bacterial genomes (Fig. 4 and Table S2). We then examined the gene arrangement in the neighborhood of 2,678 bacterial *sspBCD* modules using the sequences of *sspE* from FF75 and *sspFGH* from FF-93 as queries. The *sspBCD* genes were found to coexist with *sspFGH* in 196 strains and with *sspE* in 199 strains, accounting for 7.3% and 7.4% of the total 2,678 strains, respectively. The *sspBCD-sspFGH* modules are preferentially distributed within the genera *Rhizobiales* (40, 20.4%), *Pseudomonas* (25, 12.8%), *Escherichia* (18, 9.2%), and *Burkholderia* (10, 5.1%). In contrast, *sspBCD-sspE* is enriched in *Vibrio* (35, 17.6%), *Escherichia* (34, 17.1%), *Streptomyces* (33, 16.6%), and *Photobacterium* (13, 6.5%) (Table S2). Interestingly, *sspFGH* and *sspE* were found to be nested simultaneously within 36 additional bacterial genomes, agreeing well with the observed compatibility of the SspFGH and SspE defense mechanisms.

10.1128/mBio.00613-21.4 TABLE S2 Occurrence of *ssp* genes in 2,678 bacterial strains Table S2, XLSX file, 0.5 MB. Copyright © 2021 Wang et al. 2021 Wang et al. https://creativecommons.org/licenses/by/4.0/ This content is distributed under the terms of the Creative Commons Attribution 4.0 International license.

**FIG 5:**
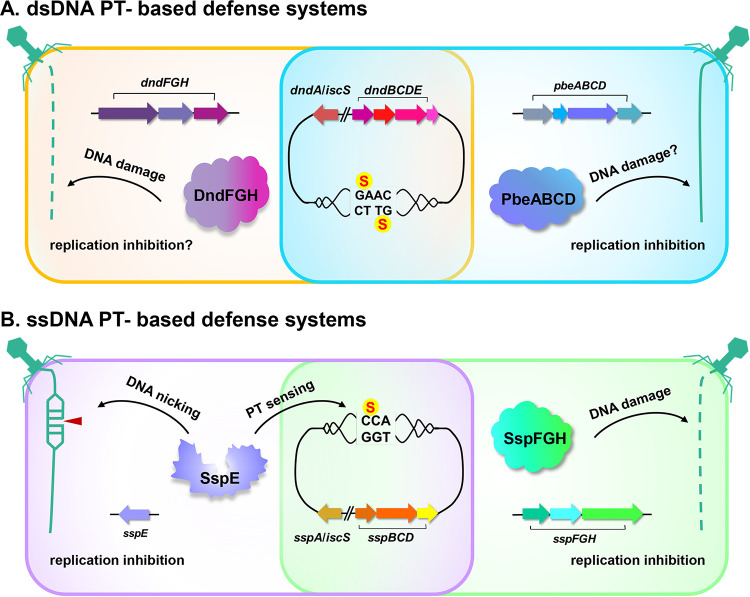
Schematic representation of the dsDNA and ssDNA phosphorothioate-based defense systems.

By concatenating the homologs of SspBCD in each bacterial strain, we built a maximum likelihood tree using MEGA X 11.0 (Fig. 4). The immediate observation was that the phylogenetic tree failed to exhibit an obvious clustering of *sspBCD* into different groups according to proximate *sspFGH*, *sspE*, both, or none, indicating potential horizontal gene transfer events (Fig. 4). For instance, Burkholderia gladioli Co14 and Burkholderia gladioli Coa14 harbor *sspBCD*-*sspFGH* and *sspBCD-sspE* cassettes, respectively, while Burkholderia gladioli pv. *gladioli* KACC 11889 possessed only the orphan *sspBCD* PT-modifying component (Fig. 4). Similarly, the distribution of *sspBCD*, *sspBCD-sspFGH*, and *sspBCD-sspE* across *Pseudomonas* was also patchy (Fig. 4). It was surprising to observe that up to 2,211 of 2,678 (83.9%) examples of *sspBCD* were not accompanied by *sspFGH* or *sspE*, which raised two possibilities: (i) there are more diversified restriction counterparts capable of constituting defense barriers with SspBCD, and (ii) some Ssp systems occur as bona fide orphan SspBCD systems lacking restriction cognates. Based on our previous results showing that solitary DNA PT modification catalyzed by DndABCDE has evolved additional functions, such as the epigenetic control of gene expression, maintenance of cellular redox homeostasis, and environmental fitness (20, 25), we propose that PTs catalyzed by solitary SspBCD systems might also act as versatile participants in multiple cellular processes resembling that of dsDNA PTs governed by solitary DndABCDE modules. Collectively, our results established a new type of SspABCD-SspFGH defense barrier with different genetic organization from that of SspA-E or DndA-H, which highlights the diversity of PT-based defense mechanisms and expands our knowledge of the arsenal of defensive measures (Fig. 5).

## DISCUSSION

In this study, we identified a new type of ssDNA PT-based SspFGH defense system exhibiting different genetic organization and phenotypic behavior from those of SspABCD-SspE and DndABCDE-DndFGH, which expands our knowledge about the diversity of gene contents and PT-based defense mechanisms. Although both were coupled with the PT-modifying component SspABCD, SspFGH and SspE responded differently upon phage infection. SspE exerts antiphage activity only in the presence of DNA containing the 5′-C_PS_CA-3′ PT modification, because sequence-specific PT is essential to stimulate SspE GTPase activity, rendering SspE a PT-sensing defense barrier (12). In PT-deficient mutants, SspE remains inactivated, leading to no apparent DNA damage or growth impairment (12). In contrast, SspFGH targets non-PT-modified DNA indiscriminately, causing DNA damage in PT-lacking host DNA and impaired cell growth. SspFGH-mediated DNA damage and phenotypic alterations resemble those arising from unrestrained DndFGH in DndABCDE-deficient mutant cells. Although SspFGH and DndFGH share no significant protein sequence identity and pair with ssDNA PT and dsDNA PT modifications, respectively, SspF and DndF both possess the Walker A motif, and DndH and SspH show some similarities to DNA translocases, e.g., FstK, implying that SspFGH and DndFGH likely share characteristics in the self/nonself discrimination mechanism.

It is interesting to observe that the 5′-C_PS_CA-3′ PT modification can simultaneously couple with both SspFGH and SspE to prevent phage infection. In canonical DNA methylation-based restriction-modification systems, the consensus sequences are nearly completely methylated by methyltransferase to prevent self-restriction from the accompanying restriction endonuclease. However, this situation is quite different for DNA PT modifications, which were found to occur heterologously at a specific site even in the presence of active DndFGH or SspE (18, 26). Despite cell-to-cell heterogeneity, the 5′-C_PS_CA-3′ PT modification can be recognized as self-DNA by SspFGH, SspE, and their combination. This raises two possibilities: (i) PT-based defense systems involve controlled restriction activity, and (ii) more elements are involved in enabling SspFGH and SspE to discriminate self from nonself DNA and exert restriction functions, such as PT density along individual DNA molecules.

Notably, SspFGH and SspE are compatible, and their combination led to additive gains in the level of phage resistance. SspE was found to nick invasive foreign DNA and consequently inhibit phage DNA replication, while SspFGH also exerted antiphage activity by introducing damage to phage DNA. One can envisage a scenario in which phage genomes that enter the host cell face the actions of two restriction systems simultaneously to prevent them from completing their infective cycle, leading to increased phage resistance. This agrees well with the observation that 36 strains were found to possess SspFGH and SspE simultaneously. However, not all combinations of defense modules are additive. The abortive phage infection gene *abiE* in conjugation *abiF* appeared to have no significantly enhanced resistance against phages in Lactococcus lactis (27). These data highlight the complicated interactions of different modes of defense mechanisms and provide further evidence for taking full advantage of the benefits of their combinations.

We previously found that phage T5 is capable of evading restriction by SspE. When incubated with phage T5 DNA *in vitro*, SspE can still introduce damage to T5 DNA, ruling out the possibility that phage T5 escapes restriction by SspABCD-SspE via DNA chemical modifications (12). While SspFGH exerts a different mode of antiphage action from SspE, SspFGH was also found to be incapable of resisting the invasion of phage T5. We presume that the insensitivity of T5 to PT-based SspFGH and SspE was attributed to its unusual DNA injection steps. Unlike most phages, T5 transfers 7.9% of its DNA, namely, the first-step-transfer (FST) DNA, to a host cell to degrade the host DNA, inhibit DNA methylation, restrict insensitivity, and synthesize one or more proteins required for complete transfer of the remaining portion of DNA (28). In addition to SspE and SspFGH, phage T5 has been found to be unsusceptible to defense by restriction endonucleases, including EcoRI, EcoK, and EcoPI, despite the presence of restriction sites in its genome (29).

In summary, we have identified a new type of PT-based defense system in which SspABCD catalyzes ssDNA PT modification and SspFGH introduces damage to non-PT DNA and consequently impairs phage DNA replication. Given the wide distribution of PT-based SspFGH defense systems and their coexistence with SspE within some bacteria, our study highlights the diversity of PT-based antiphage mechanisms and expands our knowledge about the arsenal of defensive measures.

## MATERIALS AND METHODS

### Bacterial strains, plasmids, phages, and growth conditions.

All the bacterial strains, plasmids, and phages used in this study were listed in Table S1 in the supplemental material. *Vibrio* strains were grown in tryptic soy broth (TSB) or on agar plates at 28°C as previously described (12). E. coli strains were cultured in Luria-Bertani (LB) broth or on agar plates at 37°C. E. coli cells expressing *Vibrio* genes were cultured at 28°C. When necessary, selective antibiotics (100 μg/ml ampicillin or 25 μg/ml chloramphenicol) were added to the medium.

10.1128/mBio.00613-21.3 TABLE S1 Strains, phages, and plasmids used in this study Table S1, DOCX file, 0.03 MB. Copyright © 2021 Wang et al. 2021 Wang et al. https://creativecommons.org/licenses/by/4.0/ This content is distributed under the terms of the Creative Commons Attribution 4.0 International license.

### Plaque spot assays.

After overnight incubation, the JM109 cells were diluted in LB broth at a ratio of 1:100 (vol/vol), and the optical density at 600 nm (OD_600_) was monitored until it reached 0.6. An aliquot of the cell culture (500 μl) was mixed with 5 ml of molten 0.75% top agar and spread on a preprepared 1.5% LB agar plate to grow a bacterial lawn. Tenfold serial dilutions of phages were prepared in SM buffer (100 mM NaCl, 8 mM MgSO_4_ and 50 mM Tris-HCl, pH 7.5), and 2 μl of each dilution was spotted onto the cell lawn. Images of plaques were taken after incubation at 28°C for 16 h.

### Efficiency of plaquing.

Phage PFU were quantified using the double-agar-layer technique by mixing 100 μl of phage with 200 μl of bacterial culture (OD_600_ of 0.6). Individual plaques were counted after 16 h of growth at 28°C. EOP was determined as PFU per millilter (JM109 expressing *iscS-sspBCD-sspFGH* module and derivatives)/PFU per milliliter (JM109 containing empty vectors).

### Adsorption assay.

E. coli JM109 expressing or lacking the *iscS-sspBCD-sspFGH* genes was grown in LB broth at 28°C to an OD_600_ of 0.4. Then, phage T4 was added at a multiplicity of infection (MOI) of 1. Bacterial liquid mixed with phage was cultured with shaking at 28°C. An aliquot of 1 ml culture was taken at 1, 5, 10, 15, 20, and 25 min after infection. Then, the bacteria were centrifuged at 10,000 × *g* for 1 min to precipitate. Phages that had not yet been taken up by the cells were taken from the supernatant, and the number of phages was measured by the plate experiment. The percentage of extracellular phage T4 was calculated assuming the initial titer of T4 (without added JM109 cells) to be 100%.

### TUNEL assay.

TUNEL assay was applied for detection of DNA damage using the one-step TUNEL apoptosis assay kit (Beyotime, China) according to the manufacturer’s instructions. In brief, the JM109 cells were collected by centrifugation at 13,000 × *g* for 2 min, washed once with phosphate-buffered saline (PBS), resuspended in 1 ml of fixing solution (4% paraformaldehyde in PBS) and incubated for 40 min at 28°C. Cells were washed three times with PBS and then pelleted again by centrifugation, resuspended in 1 ml of permeabilization solution (0.3% Triton X-100 in PBS), and incubated for 40 min at 28°C. At the end of the incubation, cells were resuspended in 50 μl of the TUNEL reaction mix from the kit and incubated at 37°C in the dark for 70 min. The reaction mix contains TdT, FITC-labeled dUTP, and buffers. Cells were washed three times with PBS and then resuspended in 500 μl of PBS for flow cytometry.

### Southern blot assays.

After overnight incubation at 28°C, the JM109 cells containing *iscS-sspBCD-sspFGH* or empty vectors were diluted in LB broth at a ratio of 1:100 (vol/vol) and cultured to an OD_600_ of approximately 0.6, followed by infection by phage T4 at an MOI of 6. Total DNA was extracted from infected cells collected throughout the phage infection time course using the chloroform-phenol method. A total of 2 μg intracellular DNA was digested by PacI for 6 h followed by electrophoresis on a 1% agarose gel. The gel was transferred to an Amersham Hybond-N+ nylon membrane (GE Healthcare) in 20× SSC buffer (0.3 M trisodium citrate and 3 M NaCl, pH 7.0). Subsequent procedures, including blotting, washing, and signal detection, were performed using the DIG-High Prime DNA labeling and detection starter kit II (Roche) according to the manufacturer’s protocol.

### Fluorescence microscopy.

JM109 cells containing different plasmids were collected by centrifugation (5,000 × *g*, 5 min), washed three times with PBS (pH 7.4), and resuspended in an appropriate volume of PBS. Ten microliters of the cell suspension was spread on a glass slide, dried at room temperature, and fixed with drops of methanol for 5 min. After fixing, the slide was washed six times with tap water and then dried completely at room temperature. Then, 10 μl of poly-l-lysine (5 mg/ml in distilled water) was applied to fix cells tightly to the slide. DAPI solution (10 μl of 4′,6-diamino-2-phenyl-indole at a concentration of 5 mg/ml in PBS buffer) was spread over the sample and incubated for 5 min at room temperature. Ten microliters of FM4-64 (2 mg/ml in distilled water) was subsequently dropped onto the cell sample. The cells were viewed with a Nikon A1 confocal system. DAPI and FM4-64 were observed using excitation wavelength 405 nm (Ex405) and Ex561 laser filters, respectively.
