# Supplementary figures and images for: SspABCD-SspFGH Constitutes a New Type of DNA Phosphorothioate-Based Bacterial Defense System
Source: mBio. 2021 Apr 27;12(2):e00613-21. doi: 10.1128/mBio.00613-21 (PMC8092258; doi:10.1128/mBio.00613-21)

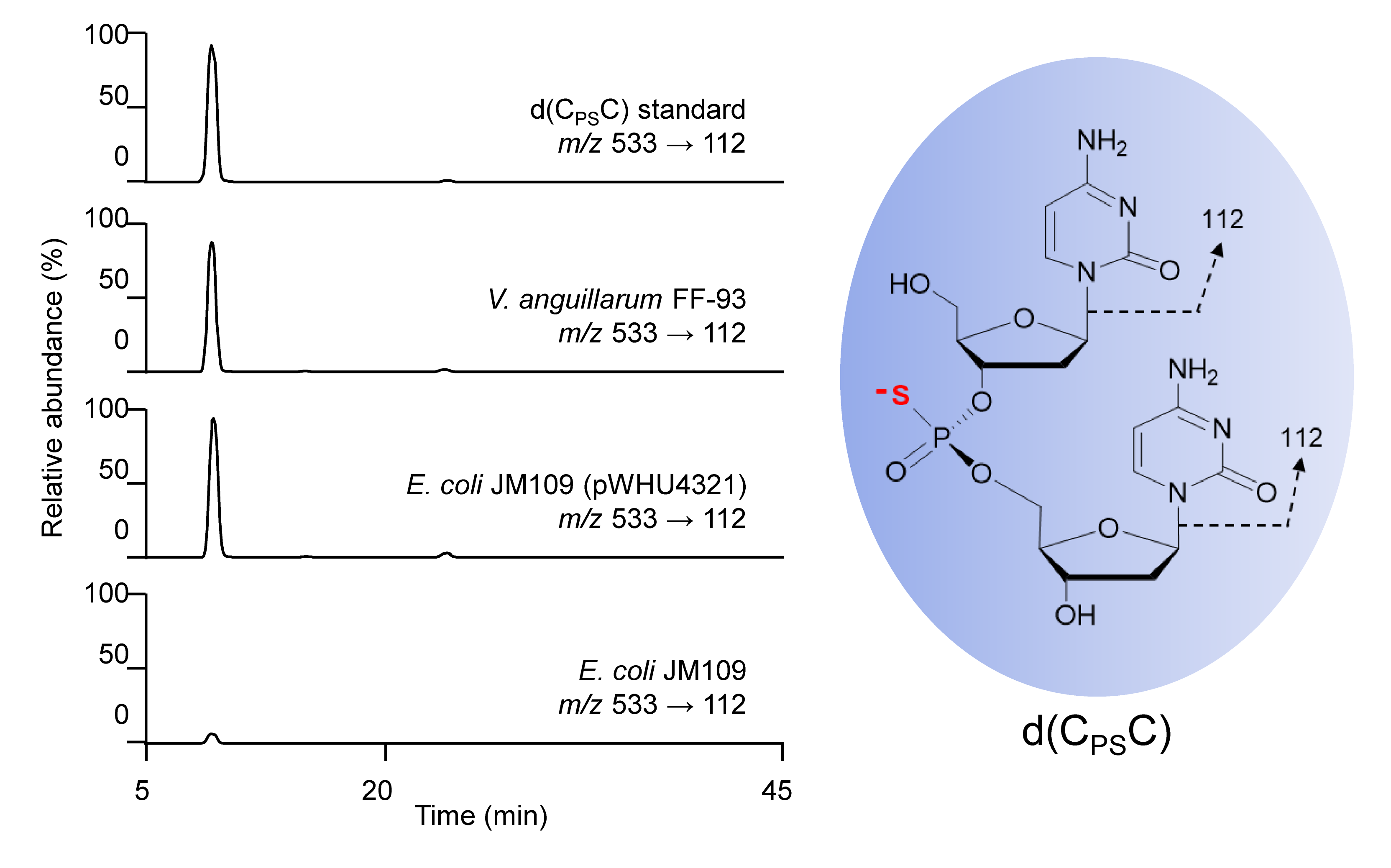

Supplement: FIG S1 [file mBio.00613-21-sf001.tif]

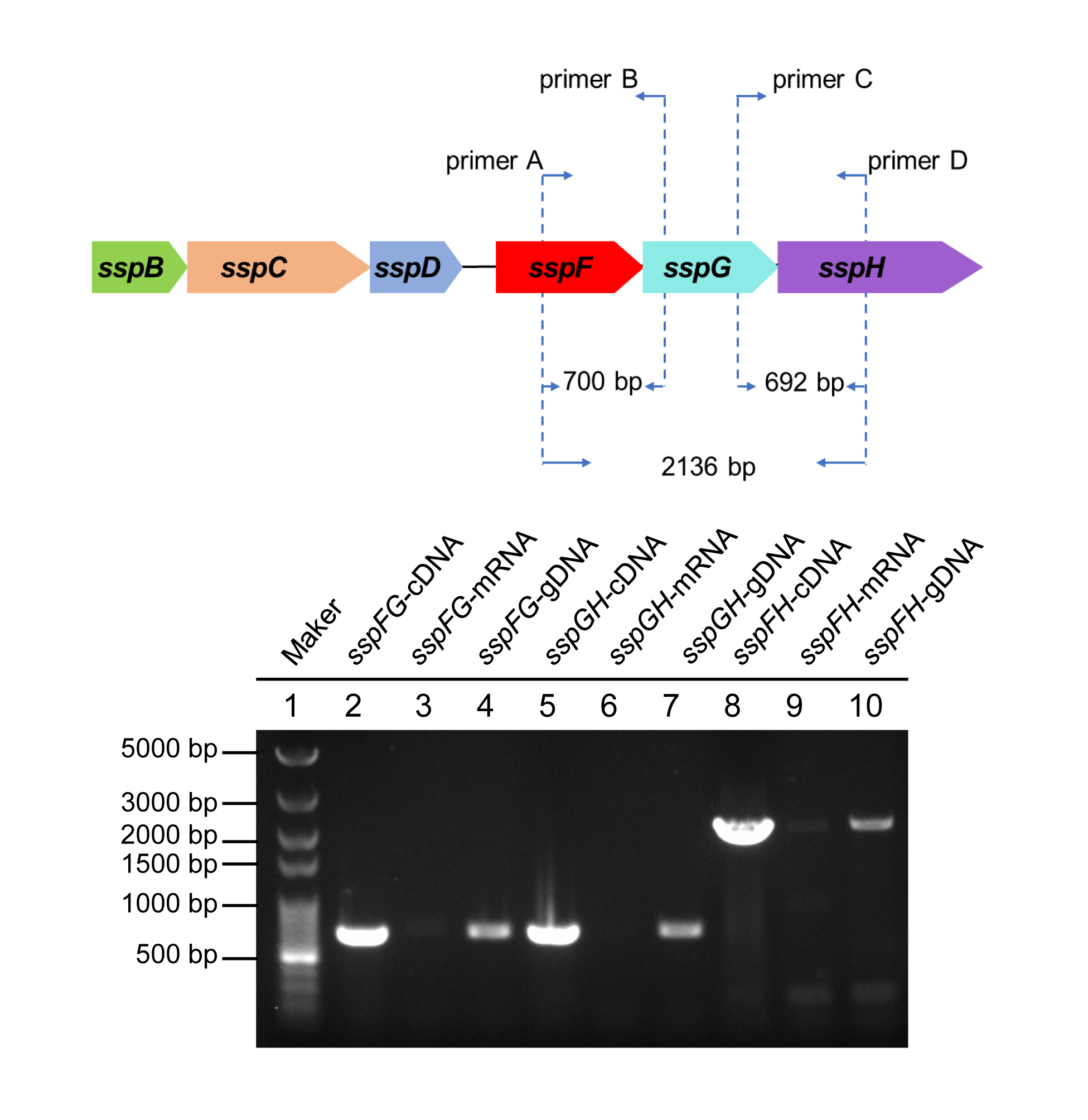

Supplement: FIG S2 [file mBio.00613-21-sf002.tif]
