# Supplementary material for: SspABCD-SspFGH Constitutes a New Type of DNA Phosphorothioate-Based Bacterial Defense System
Source: mBio. 2021 Apr 27;12(2):e00613-21. doi: 10.1128/mBio.00613-21 (PMC8092258; doi:10.1128/mBio.00613-21)
Supplement: TABLE S1 [file mBio.00613-21-st001.docx]

**TABLE S1. Strains, phages and plasmids used in this study**

| **Strains, plasmids and phages** | | **Characteristics** | **Source or reference** |
| --- | --- | --- | --- |
| ***Vibrio*** |  | |  |
| *V. anguillarum* FF-93 | SspBCD-SspFGH, 5’-C_PS_CA-3’, GenBank: AJYT00000000.2 | | (1) |
| *V. cyclitrophicus* FF75 | SspBCD-SspE, 5’-C_PS_CA-3’, GenBank: ATLT01000000 | | (1, 2) |
|  |  | |  |
| ***E. coli*** |  | |  |
| JM109 | endA1 glnV44 thi-1 relA1 gyrA96 recA1 mcrB^+^ Δ(lac-proAB) e14- [F’ traD36 proAB^+^ lacl^q^ lacZΔM15] hsdR17(r_k_^-^m_k_^+^) | | TransGen Biotech |
| **Plasmids** |  | |  |
| pBluescript II SK(+) | Cloning vector, Amp^r^ | | (3) |
| pACYC184 | Cloning vector, Cm^r^ | | (4) |
| pWHU4321 | SK(+) derivative with a 5.3-kb KpnI–BamHI fragment carrying *sspBCD* and a 1.5-kb SacI-NotI fragment carrying *iscS* from FF-93 | | This work |
| pWHU4322 | pACYC184 derivative with a 9.0-kb XbaI-SalI fragment carrying *sspFGH* from FF-93 | | This work |
| pWHU4324 | pACYC184 derivative with a 2.8-kb XbaI-SalI fragment expressing *sspE* from 3234/A | | This work |
| pWHU4325 | pWHU4322 derivative with a 2.8-kb KasI-NruI fragment carrying *sspE* from 3234/A | | This work |
| pWHU4326 | pACYC184 derivative with a 7.9-kb XbaI-SalI fragment carrying *ΔsspF*-*sspGH* from FF-93 | | This work |
| pWHU4327 | pACYC184 derivative with an 8.0-kb XbaI-SalI fragment carrying *ΔsspG-sspFH* from FF-93 | | This work |
| pWHU4328 | pACYC184 derivative with a 4.7-kb XbaI-SalI fragment carrying *ΔsspH-sspFG* from FF-93 | | This work |
| pWHU4330 | SK(+) derivative with a 5.4-kb KpnI-BamHI fragment carrying *sspBCD* from 3234/A | | This work |
| **Phages** |  | |  |
| T4 | *Myoviridae*, lytic, dsDNA | | (5) |
| T1 | *Siphoviridae*, lytic, dsDNA | | (5) |
| JMPW1 | *Siphoviridae*, lytic, dsDNA | | (6) |
| T5 | *Siphoviridae*, lytic, dsDNA | | (5) |
| EEP | *Siphoviridae*, lytic, dsDNA | | (7) |

**References**

1. Cordero OX, Wildschutte H, Kirkup B, Proehl S, Ngo L, Hussain F, Le Roux F, Mincer T, Polz MF. 2012. Ecological populations of bacteria act as socially cohesive units of antibiotic production and resistance. Science 337:1228-31.

2. Xiong X, Wu G, Wei Y, Liu L, Zhang Y, Su R, Jiang X, Li M, Gao H, Tian X, Zhang Y, Hu L, Chen S, Tang Y, Jiang S, Huang R, Li Z, Wang Y, Deng Z, Wang J, Dedon PC, Chen S, Wang L. 2020. SspABCD-SspE is a phosphorothioation-sensing bacterial defence system with broad anti-phage activities. Nat Microbiol 5:917-928.

3. Alting-Mees MA, Short JM. 1989. pBluescript II: gene mapping vectors. Nucleic Acids Res 17:9494.

4. Chang AC, Cohen SN. 1978. Construction and characterization of amplifiable multicopy DNA cloning vehicles derived from the P15A cryptic miniplasmid. J Bacteriol 134:1141-56.

5. Demerec M, Fano U. 1945. Bacteriophage-Resistant Mutants in Escherichia Coli. Genetics 30:119-36.

6. Shen M, Zhu H, Lu S, Le S, Li G, Tan Y, Zhao X, Shen W, Hu F, Wang J. 2016. Complete Genome Sequences of T1-Like Phages JMPW1 and JMPW2. Genome Announc 4.

7. Li S, Liu L, Zhu J, Zou L, Li M, Cong Y, Rao X, Hu X, Zhou Y, Chen Z, Hu F. 2010. Characterization and genome sequencing of a novel coliphage isolated from engineered Escherichia coli. Intervirology 53:211-20.
